# Supplementary material for: CycleGAN for interpretable online EMT compensation
Source: Int J Comput Assist Radiol Surg. 2021 Mar 14;16(5):757–65. doi: 10.1007/s11548-021-02324-1 (PMC8134291; doi:10.1007/s11548-021-02324-1)
Supplement: Supplementary file 1 — Supplementary material 1 (pdf 83 KB) [file 11548_2021_2324_MOESM1_ESM.pdf]

Supplemental Material: Online Positional Error Compensation for EMT using CycleGAN

Henry Krumb Dhritimaan Das Romol Chadda
Anirban Mukhopadhyay

November 3, 2020

1 Variance of x-, y- and z-axes

In our paper (Methods section), we state that there is more variance along the z-axis than in the xy-plane. This assumption is essential for our method, as the CycleGAN-approach only changes the z, q and rotational components, leaving the x- and y-components untouched. We therefore want to substantiate our claim, by providing standard deviations of the different DoF in Table 1. To compute these standard deviations, all points were grouped by their associated ground-truth point, and then mean-centered in their respective groups. After mean-centering, groups were merged again to compute standard deviation. As Table 1 shows, standard deviation is about one order of magnitude higher on the z-axis than on the xy-plane.

Dataset	σ_x	σ_y	σ_z
laboratory	0.067	0.082	**0.137**
c-arm 7 cm	0.263	0.158	**1.465**
c-arm 9 cm	0.237	0.128	**1.436**
c-arm 12 cm	0.199	0.104	**1.215**

Table 1: Standard deviations of individual positional components for evaluation and lab datasets.

2 Phantom Experiment

Please find attached to this submission an animated GIF showing the retraction of the EMT sensor in our aortic phantom. The animation directly corresponds to the picture in our manuscript (Figure 6). Please note that the retraction did not happen in real time, but in discrete steps of 8 mm.
